# Bladder exstrophy in adulthood

**DOI:** 10.4103/0970-1591.40609

**Published:** 2008

**Authors:** R. B. Nerli, G. V. Kamat, S. S. Alur, Ashish Koura, Vikram Prabha, S. S. Amarkhed

**Affiliations:** Department of Urology, KLES Kidney Foundation, KLES Prabhakar Kore Hospital and MRC, Nehru Nagar, Belgaum, Karnataka, India

**Keywords:** Adult, augmentation ileocystoplasty, bladder reconstruction, epispadias, exstrophy bladder

## Abstract

**Background:**

We report our experience with the treatment of classic exstrophy of the bladder in a small series of seven adult males. There are very few documented cases of adults presenting with exstrophy of bladder in literature.

**Materials and Methods:**

Adult males presenting with classic exstrophy of the bladder and complete epispadias underwent detailed evaluation including psychological assessment and counseling. All were explained regarding the surgical procedure and informed about the need for self-catheterization. Prior to 2002 all patients underwent cystectomy of the existing bladder plate, with creation of catheterizable modified Mainz pouch. Since 2002 patients were assessed for bladder reconstruction with augmentation ileocystoplasty, bladder neck reconstruction, and abdominal wall closure.

**Results:**

Seven adult males with classic exstrophy of the bladder and complete epispadias who had not received any previous treatment presented to us during the period 1991-2006. Five of these underwent cystectomy with continent pouch and the remaining two underwent bladder reconstruction. All have been doing well with improved self-esteem and social interaction. Two of these have married and leading a satisfactory sexual relationship.

**Conclusions:**

Surgical correction in adults with exstrophy of the bladder greatly improves self-esteem, confidence, and social relationship.

## INTRODUCTION

Bladder exstrophy remains one of the most challenging conditions managed by pediatric urologists. Although rare, this disorder imposes significant physical, functional, social, sexual, and psychological burdens on patients and families. The incidence of bladder exstrophy has been estimated as between 1 in 10,000 and 1 in 50,000 live births.[1] However, Nelson *et al*.[2] reporting on contemporary epidemiology of bladder exstrophy in the United States showed sample results in a weighted national incidence of 2.15 bladder exstrophy cases per 100,000 live births. The male to female ratio of bladder exstrophy derived from multiple series is 2.3:1.[3] Nelson *et al*.[2] reported that males and females were roughly equally affected by exstrophy. The male to female ratio did not differ by race, insurance status, or region.[2]

Reconstructive management of exstrophy bladder is the best started in the neonatal period with good results. As the defect is obvious and definitive treatment with primary reconstruction being possible in infancy, it is rare to see an adult presenting with exstrophy of bladder.[4] When left untreated, exstrophy has malignant potential.[5] The exact incidence of patients with bladder exstrophy seeking treatment in adulthood is not documented adequately in literature. We report a series of seven patients with bladder exstrophy who were managed for the same in adulthood.

## MATERIALS AND METHODS

Adults seeking treatment for untreated exstrophy of bladder underwent a complete evaluation of genitourinary tract. Laboratory investigations included renal function tests and complete hemogram. Ultrasonography, Intravenous urography, and plain X-ray of the pelvis were done in all. Multiple random bladder biopsies were taken to look for dysplastic or neoplastic changes. All patients were informed regarding the need for surgery and counseled about the need for self-catheterization.

Prior to 2002, all patients underwent cystectomy and creation of a catheterizable Mainz pouch. The patient was taught self-catheterization and advised to practice the same 2 weeks after the surgery. After 2002, patients having a classical exstrophy were also assessed for surgical correction of bladder exstrophy. On bladder biopsies showing no evidence of dysplastic or neoplastic changes, bladder reconstruction and repair was done in two stages. The first stage included ileocytoplasty to increase the bladder capacity, Young Dees bladder neck reconstruction and a secure abdominal wall closure using flaps. The epispadias was repaired by the modified Cantwell-Ransley method at the second stage 6 months later. All these patients had their drain, suprapubic catheter and urethral stent removed at an appropriate time. The patients were advised self-clean intermittent catheterization.

Postoperatively all patients were followed up with repeated urine examinations, ultrasound examinations, and estimation of serum creatinine. All patients were counseled after surgery regarding body image, sexual function, and vocational training.

## RESULTS

During the period July 1991 to July 2006 seven adult males were treated for classic exstrophy of the bladder with complete epispadias. We studied the case records and analyzed the findings. The mean age at presentation was 19 years (17-22 years). The reasons for not having approached for treatment earlier included ignorance and poverty. All the seven patients came from rural background. At presentation five of these patients had attended the school, one had no formal education and one was planning to join the college. The reasons given by these patients for seeking treatment now were that they had become social outcasts. Incontinence and urinary smell had been their main bother. All patients had low self-esteem, poor confidence levels, and poor social relations. All these patients had pressurized their parents and relatives to seek treatment for their condition. All patients had normal mental growth with normal IQ. Performance status in school of the six patients who attended school was good.

**Figure 1:**
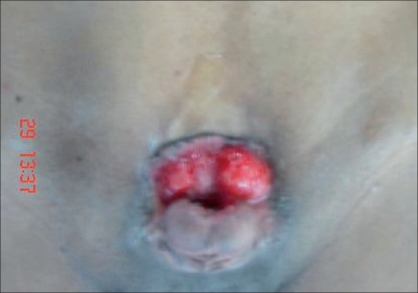
Adult exstrophy before surgery

**Figure 2:**
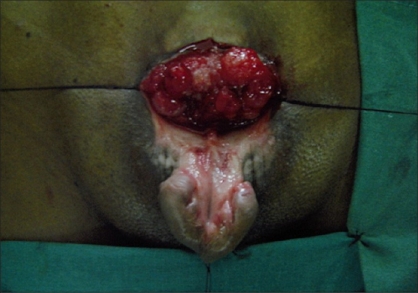
Adult exstrophy before surgery

**Figure 3:**
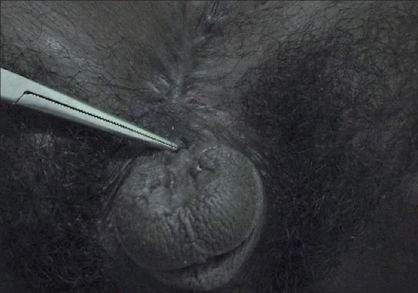
Epispadias before repair

**Figure 4:**
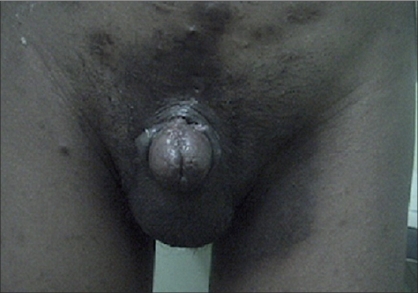
Epispadias after repair

**Figure 5:**
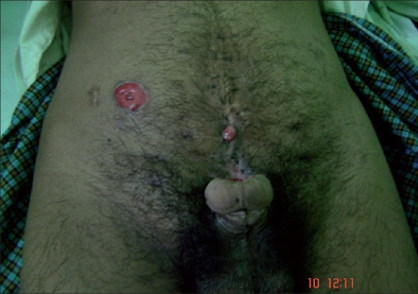
Adult exstrophy after continent diversion

Various social organizations, insurance companies, and charitable institutions supported these patients financially. Four patients presenting prior to 2002 underwent cystectomy with creation of catheterizable modified Mainz pouch. Postoperatively all these patients did well. Self-intermittent catheterization of the abdominal stoma was started from the second week. All these patients underwent epispadias repair 6 months later. There were no intraoperative or postoperative complications. All these four patients have been on follow-up ranging from 6 to 15 years. The patients needed to catheterize once in 4-5 h. The Mainz pouch capacity ranged from 350 to 450 cc. All patients have remained dry during the daytime and only one patient has occasional nighttime urinary leak. The patients have improved in their self-esteem, confidence, and social interaction. Two of these four have been married and have satisfactory erections and sexual relations. The remaining two also have adequate erections.

Since 2002 two patients underwent bladder reconstruction in the first stage which included, augmentation ileocystoplasty, Young Dees bladder neck reconstruction and a secure abdominal wall closure and one patient underwent cystectomy with creation of catheterizable modified Mainz pouch. The patient who underwent cystectomy was the one who had a very small bladder template. All these three patients underwent epispadias repair 6 months later. All these three patients were on clean self-intermittent catheterization. These three patients have similarly improved in their outlook, self-esteem, confidence, and social interaction following surgery. All these three patients have adequate erections. All these three patients are on regular follow-up. The two patientsx who underwent bladder reconstruction had a near normal appearing bladder on cystoscopy a year later. These two patients are on regular follow-up with ultrasonography and urinary examination to monitor the bladder. Interval endoscopic assessment of the bladder has been advised to these patients.

The abdominal wall closure was achieved primarily in all five patients who underwent cystectomy followed by catheterizable Mainz pouch. No flaps were necessary in any of these patients. Two patients who underwent bladder reconstruction, required flaps to secure abdominal wall closure. We have used crossed rectus flaps and rectus sheath flaps for securing abdominal wall closure.

Wound complications occurred specifically in patients with bladder reconstruction which included wound infection and partial wound dehiscence not requiring any active surgical intervention.

All the seven patients are satisfied with the cosmetic results of the procedures. All the seven patients are dry during the daytime; however, only one patient undergoing Mainz pouch reconstruction, complained of occasional wetting in the nighttime. There was no deterioration of renal function seen in any of these patients during follow-up. Only one patient had two episodes of symptomatic urinary tract infection during follow-up. Presently, three of these patients are working in the fields, one is attending college and the remaining three are engaged in small business.

## DISCUSSION

Neonatal total reconstruction of bladder exstrophy-epispadias complex is the treatment of choice. Primary reconstruction is increasingly performed with good results. Adults presenting with exstrophy is very rare, as the obvious deformity with leaking urine cannot go unnoticed and can lead to difficulties due to upper tract dysfunction, difficulty in secure abdominal closure, and malignant potential of the bladder remnant.

Historically, ureterosigmoidostomy was the first form of diversion to be popularized for patients with exstrophy. Although the initial series was associated with multiple metabolic problems, results improved markedly with newer techniques of reimplantation.[4] Ureterosigmoidostomy is favored by some because of the lack of an abdominal stoma. One should be cautious before advising this form of diversion and is certain that anal continence is normal and after the family has been made aware of the potential serious complications, including pyelonephritis, hyperkalemic acidosis, rectal incontinence, ureteral obstruction, and delayed development of malignancy.[6,7]

Matsuda *et al*.[8] reported a case of a bladder exstrophy in an adult female, treated with cystectomy, construction of a Kock's continent ileal reservoir, and closure of the abdominal fascial defect using alloplastic material. The Kock's ileal reservoir improved the quality of her life not only physically, but also mentally by affording her urinary continence. The Kock's ileal continent reservoir overcomes the problems associated with ureterosigmoidostomy, namely, recurrent infections, pyelonephritis, metabolic and electrolyte disturbances, fecal incontinence, and colonic neoplasia.[7]

Gulati *et al*.[9] reported management of bladder exstrophy in adulthood. Two adult females presenting with untreated bladder exstrophy underwent cystectomy and modified Mainz pouch. The abdominal wall defect was closed primarily. Both these patients had improved quality of life and renal function. Stein and associates[4,10] treated a group of 128 patients with bladder exstrophy-epispadias with the Mainz technique for ureterosigmoidostomy. D'Elia *et al*.[11] reported on 26 patients with the exstrophy-epispadias complex who showed excellent continence rates with long-term upper tract preservation when compared with standard ureterosigmoidostomy.

Ozdiler *et al*. [12] reported a 49-year-old female who presented with exstrophy of bladder. Quattara *et al*.[13] also reported a 39-year-old male who had presented with exstrophy of bladder.

In our series five patients underwent cystectomy with creation of modified Mainz pouch, four of these underwent this procedure prior to 2002. Due to rarity of these cases, patients were offered cystectomy to remove the bladder plate as well as creation of modified Mainz pouch to render the patient continent. The main concern of these patients at the time of presentation was urinary incontinence, urinary smell, and social ostracism. None of these patients showed any interest regarding management of epispadias component at this time primarily because of ignorance, financial constraint, and the concern for urinary incontinence. However, all five patients showed an interest in epispadias repair once continence was achieved. Second, these patients being in adolescent/young adulthood started appreciating erections and felt the need for sexual relationship/marriage. The two patients who underwent augmentation cystoplasty with bladder neck reconstruction and abdominal wall closure were offered management of epispadias as a staged procedure by us.

Pathak *et al*.[14] reported the treatment of classic exstrophy of bladder in four adult patients using ileocystoplasty, bladder neck reconstruction, and abdominal wall closure with flaps. Three patients were treated in two stages. The first stage included ileocystoplasty, bladder neck reconstruction, and abdominal wall closure with the use of flaps. The epispadias was repaired in the second stage. In one patient, the reconstruction was completed in a single stage. All patients were continent at the last follow-up visit, with three using self-catheterization and one voiding spontaneously. After surgery, all demonstrated improved social interaction. They concluded that vesical preservation with primary reconstruction of bladder exstrophy in adults was safe and feasible in the absence of significant histologic changes in the bladder mucosa. Pathak *et al*.[14] believe that although the bladder mucosa will be, inflamed before closure, in the absence of dysplastic changes, it is suitable for bladder neck reconstruction and augmentation and the inflammation subsides with proper closure. Pathak *et al*.[14] also opined that the patients demonstrated increased self-confidence and improved social interaction as a result of being continent and having a normally placed meatus and a cosmetically improved phallus following surgery.

Gearhart[15] expressed that it was important to save and use bladder template for it had two advantages. First, using bladder template meant taking less bowel and second, if ureteral reimplantation was necessary, the bladder template was a much better substrate for reimplantation than a bowel wall. Though we have not compared the patients undergoing the two different procedures, we feel that the patients get well adjusted psychosexually and psychologically primarily because they become dry in both these procedures.

## CONCLUSIONS

Bladder exstrophy is very rare in adulthood and presents with urinary incontinence and social stigma. Patients undergoing cystectomy with creation of a continent pouch or bladder reconstruction have improved self-confidence, self-esteem, and social interaction after surgery. Long-term psychosexual and psychologic follow-up in these individuals show that these patients are well adjusted primarily because they are continent and remain dry.
